# Empowering tuberculosis genomic surveillance in Limpopo, South Africa through capacity building

**DOI:** 10.3389/fpubh.2025.1567382

**Published:** 2025-09-12

**Authors:** Vukosi Treasure Makondo, Kabelo Gabriel Kaapu, Felicia Wells, Abhinav Sharma, Molebogeng Ruth Lekalakala-Mokaba, Robin Warren, Emilyn Costa Conceição, Ivy Rukasha

**Affiliations:** ^1^Division of Medical Microbiology, Department of Pathology, School of Medicine, University of Limpopo, Polokwane, South Africa; ^2^South African Medical Research Council Centre for Tuberculosis Research, Division of Molecular Biology and Human Genetics, Faculty of Medicine and Health Sciences, Stellenbosch University, Cape Town, South Africa; ^3^Department of Microbiology, National Health Laboratory Service, Polokwane, South Africa

**Keywords:** *Mycobacterium tuberculosis*, drug resistance, Limpopo, genomic surveillance, multi-drug resistance, capacity building

## Abstract

**Introduction:**

Limpopo, a predominantly rural province in South Africa, faces significant challenges in the management of tuberculosis (TB) due to its high mobility and limited healthcare infrastructure. This study aims to improve the genomic surveillance of TB in Limpopo through capacity building initiatives.

**Methods:**

A comprehensive training program was implemented that focuses on both theoretical and practical aspects of TB research, including whole genome sequencing (WGS) and bioinformatics. Sputum samples from 232 patients diagnosed with pulmonary TB were collected, with 30 isolates selected for WGS analysis. The MAGMA bioinformatics pipeline was used for genomic analysis, identifying drug resistance mutations and phylogenetic relationships.

**Results:**

Of the 28 *Mycobacterium tuberculosis* (*Mtb*) isolates analyzed, 53.6% were females, with a median age of 39 years. The isolates predominantly belonged to Lineage 4 (53.6%) and Lineage 2 (35.7%). High levels of drug resistance were observed, with 100% of isolates resistant to rifampicin and 61% resistant to isoniazid. In particular, 54% of the isolates were resistant to fluoroquinolones (FLQs) and 18% showed resistance to bedaquiline (BDQ). Phylogenetic analysis revealed two distinct clusters, indicating localized and interdistrict transmission.

**Conclusion:**

The study highlights the genetic diversity and drug resistance patterns of *Mtb* in Limpopo, highlighting the need for continued genomic surveillance and tailored public health interventions. Capacity building efforts have laid the groundwork for improved TB diagnosis and surveillance in this strategic region.

## Introduction

Limpopo, located in northern South Africa, is the country’s fifth most populous province, contributing approximately 10% of its over 60 million people. Despite its size, it is one of South Africa’s poorest regions, primarily rural (80%) and lacking adequate infrastructure and public services, especially healthcare. The province is central to the development of South Africa’s disease trends, as it serves as a major road gateway between South Africa and other African nations bordering Botswana, Mozambique and Zimbabwe. The province has few mining districts, as well as an agricultural area of fruits and game with a significant migrant and seasonal labor population. High mobility across borders due to the frequent movement of people and animals across these international boundaries and within provinces can influence infectious diseases, such as tuberculosis (TB), in this region (1).

South Africa faces a significant challenge with tuberculosis. In 2022, the country had a TB incidence rate of 468 per 100,000 people, and it is classified on three of the World Health Organization’s (WHO) priority lists: high-burden TB, multidrug-resistant TB (MDR-TB), and HIV/TB co-infection (2). However, there are regional variations within South Africa. According to the National Institute for Communicable Diseases (NICD) on microbiologically confirmed pulmonary TB, Limpopo province demonstrated a significant decrease in TB incidence, dropping from 127 to 34 cases per 100,000 people between 2021 and 2022.^1^

Whole-genome sequencing (WGS) has proven to be valuable in TB surveillance, aiding in understanding transmission patterns through genomic analysis of *Mtb* strains and guiding traditional contact tracing efforts. WGS also offers a rapid and comprehensive identification of drug resistance mutations, including newer drugs such as bedaquiline (BDQ) and delamanid, which lack reliable phenotypic drug susceptibility tests (pDST) (3). This enables precise classification of strain into lineages and sublineages. However, the challenges in expanding WGS in low and middle-income countries (LMIC) such as South Africa include infrastructure limitations and a shortage of skilled personnel (4).

The genetic diversity of *Mtb* in Limpopo remains largely unknown due to limitations in the methods used in previous research. A single study conducted in the province employed spoligotyping, which offered low resolution and therefore did not capture the full extent of genetic variation (5). A multicenter national study conducted to assess BDQ resistance in patients treated with rifampicin-resistant TB (RR-TB) used WGS for baseline samples, excluding the 11 isolates susceptible to TB from Limpopo (6).

Implementing WGS in Limpopo is crucial to assist in the precise diagnosis and surveillance of drug-resistant (DR)-TB in a strategic frontier region. Based on the lack of *Mtb* WGS data from Limpopo, our aim was to explore the genetic characteristics of *Mycobacterium tuberculosis* (*Mtb*) isolated in Limpopo within a capacity building initiative in South Africa.

## Materials and methods

### Capacity building training framework

The training program focused on both theoretical and practical aspects of TB research, with hands-on experience in laboratory and computational methods. It was attended by two TB research students from the University of Limpopo and other academics from Stellenbosch University, South Africa. Specific activities are described in Supplementary Table 1. The samples were collected and processed at the Polokwane National Laboratory Health Service and sent to Stellenbosch University for the practical portion of the course. For bioinformatics practice and analysis, Google Cloud was used.

### Sample characteristics and processing

Sputum samples were collected from patients diagnosed with pulmonary tuberculosis who attended clinics and hospitals in Limpopo. From a collection of 232 *Mtb* isolates obtained between 1 January 2021 and 31 December 2023, stored in the NHLS Polokwane repository, 30 isolates were selected for sequencing. Due to resource constraints, only 30 isolates could be sequenced. Therefore, a purposive selection approach was used to ensure representation across a spectrum of drug-resistant TB profiles, including MDR, Pre-extensively drug-resistant (pre-XDR) and extensively drug-resistant (XDR) TB cases. All isolates exhibiting resistance to BDQ and fluoroquinolones (FLQ) were prioritized for inclusion, while the remaining rifampicin-resistant (RR) and MDR-TB samples were selected based on their geographic distribution and specific resistance patterns. Demographic data associated with the samples were extracted from the laboratory request forms submitted at the time of sample collection.

The isolates were previously confirmed as DR-TB using MTBDRplus and MTBDRsl line probe assays (LPA) (Hain LifeScience, Nehren, Germany), following established protocols (7, 8). The MTBDRplus assay was used to detect resistance to first-line drugs, isoniazid (INH) and rifampicin (RIF), while the MTBDRsl assay was used to detect resistance to second-line drugs, including second-line injectables and FLQ (9, 10).

### Subculture and quality control

Cryopreserved isolates were subcultured in BD BACTEC MGIT TM automated mycobacterial detection system culture medium using growth supplement and PANTA (BD, Franklin Lakes, USA) according to the manufacturer’s information. After the instrument was culture positive, the samples remained incubated at 37 °C for an additional 2 weeks to obtain more bacterial biomass (11, 12). Quality control measures (QC) such as microscopy using the Ziehl-Nelsen staining method and blood agar culture were performed to confirm the presence of acid-fast Bacilli (AFB) and to verify the purity of the cultured, respectively. A 5 mL aliquot of each MGIT culture was transferred to a 15 mL tube and heat inactivated for 1 h at 80 °C in the Biosecurity Level 3 laboratory (BSL3).

### DNA extraction and whole genome sequencing

Heat-inactivated aliquots were centrifuged for 30 min in the Biosafety Level 2 (BSL2) laboratory to pellet *Mtb* cells. The supernatant was carefully discarded without disturbing the pellet. DNA extraction was carried out according to the InstaGene Matrix/Fast Prep kit protocol (Bio-Rad, California, USA). The high sensitivity of Qubit ds DNA was used to quantify DNA (ng/ul) (Thermo Fisher Scientific, Waltham, United States). Genomic library preparation was performed using the Illumina DNA prep kit according to the manufacturer’s instructions (Illumina, San Diego, USA), and high sensitivity of Qubit double-strand DNA was used to quantify the libraries, while Tape Station (Aglient) was used to obtain the fragment size of genomic libraries. Sequencing was performed on the Illumina MiniSeq platform using a high output cartridge targeting a coverage of 50x.

### Bioinformatics analysis

#### Quality control and variant calling

The MAGMA (Maximum Accessible Genome for *Mtb* Analysis) bioinformatics pipeline was used to analyze clinical samples of *Mtb*. The MAGMA pipeline employs a multistep process. First, low-quality reads and adapter sequences were removed using FastQC v0.11.9 9.^2^ To check the quality of the raw sequences, Trimmomatic v0.39 was used to eliminate any poor-quality reads. The high quality reads were then aligned with a reference genome of *Mtb* (H37Rv) (GenBank accession number: NC_000962.3) and sorted with SAMtools. Duplicate reads were subsequently removed using Picard Picard-tools v2.18.25^3^ to ensure variant calling accuracy as previously described (13, 14).

#### Drug resistance detection

MAGMA generated a list of resistance calls for potential drug resistance gene variants. These calls were based on GATK-filtered Single Nucleotide Polymorphism (SNP) and unfiltered INDELs to identify key drug resistance variants. MAGMA created an Excel file containing the variant classification, detection method, frequency, and WHO catalog listing. TBProfiler v5.0 was used to determine the drug resistance profile of each sample, identifying and reporting all resistance-associated variants as described in the latest WHO drug-resistant mutation catalog (8). The profiler also reported all variants with candidate resistance genes that have unknown association with resistance and variants not listed in the WHO catalog (8). These variations were then annotated using ANNOVAR to understand their potential impact on gene function and drug resistance. MAGMA also facilitated gene set enrichment analysis to identify pathways potentially associated with drug resistance. MAGMA also provided the source of annotation within the WHO catalogue 2^nd^ Edition (8).

#### Phylogenetic outputs and visualization

MAGMA created text files detailing the lineage and drug resistance characteristics of each sample. MAGMA produced two phylo-genetic trees: the “IncComplex” tree and the “ExcComplex” tree. Both trees exclude areas related to drug resistance and other variations, such as repetitive elements and highly variable gene families like PE/PPE regions, which are typically excluded from clustering and phylogenetic analyses due to their high mutation rates and alignment challenges. The IncComplex tree includes complex regions of *Mtb,* while the ExComplex tree excludes them. The ExComplex tree, which contains information about drug resistance and lineage for each sample, was uploaded to iTol.^4^ This online platform then generated a clear and annotated maximum-likelihood phylogenetic tree. Finally, *M. canettii* was used as outgroup to root the tree, establishing the evolutionary relationships between the samples. The resulting tree was visualized using iTOL, which can label phylogenetic trees with drug resistance and lineage information based on the annotation files created by TBProfiler (14). Drug resistance information was included in the tree visualization (7). The Figtree files were automatically annotated so that the tip label of samples belonging to a cluster is the same color. The presence of clusters was also visualized in the tree, one using a distance of 11 SNP and another using a cutoff of 14 SNP.

#### Cluster analysis and recent transmission detection

MAGMA performed cluster analysis by first combining the filtered SNPs into a single file. This file was then used to calculate how similar or different each pair of samples was based on their SNPs. Pairwise SNP distances between isolates were computed with and without inclusion of complex genomic regions, defined as repetitive elements, PE/PPE gene families, transposases, and phage-associated genes — all of which are typically excluded due to their high mutation rates and alignment ambiguity. ClusterPicker used the results of these calculations and ClusterPicker used them to group the samples into groups (14). Clusters identified by ClusterPicker were visualized using the generated iTol file (14). Clustered cases were used to identify recent transmission. Clusters were defined as two or more isolates with a pairwise SNP distance of ≤11 or ≤14, based on thresholds established for identifying recent and broader transmission events. A non-clustered (unique) case was defined as any case from the study population that has a unique pattern not shared by any other case. A group of samples was considered a cluster if they had 14 or fewer differences in their genomes. This included closely related samples (within households) and somewhat related samples. We selected thresholds of 11 and 14 SNPs for clustering to capture both recent transmission and broader genomic relatedness. While much of the literature uses ≤5 SNPs for recent transmission and ≤12 SNPs for broader relatedness (15, 16), other studies in high-burden or genetically diverse settings have shown that probable transmission links may persist up to 15 SNPs (17). Phylodynamic analyses further indicate that a ≤ 4 SNP cutoff captures most recent transmissions, while >12 SNPs likely excludes direct transmission (18). Given Limpopo’s high lineage diversity, cross-border migration, and potential for delayed diagnosis, the slightly broader thresholds allow for detection of extended transmission chains that may still be epidemiologically relevant.

The clustering rate was calculated as the proportion of total isolates that belonged to at least one cluster:

Clustering rate = (Number of clustered isolates ÷ Total number of isolates) × 100.

An isolate was considered clustered if it was part of a group of two or more isolates meeting the SNP distance cutoff. Isolates that did not share genomic similarity within the threshold with any other isolate were considered unique or non-clustered.

### Statistical analysis

All statistical analyses were performed with SPSS v28.1 software. Descriptive statistics were used to present the data. The Chi-square and Fisher’s exact test were used for categorical data, i.e., to determine any relationship between lineages and drug resistance and also geographical distribution. The association between the demographic characteristics of the individuals with the appearance of clustered and unclustered Mtb was examined using the odds ratio. The p-value “*p* < 0.05” was considered statistically significant.

### Ethical approval

The study was granted ethical approval by the Turfloop Research Ethics Committee (TREC/03/2024: PG). The waiver of individual patient consent was granted since the study used isolates and had no direct involvement with the patients. NHLS granted permission to access the isolates for research purposes through its Academic Affairs and Research Management System (AARMS).

## Results

Twenty-eight of 30 sequenced *Mycobacterium tuberculosis* (Mtb) isolates were included in the primary analysis. Two isolates were excluded: one was identified as nontuberculous mycobacteria (NTM), specifically *Mycobacterium europaeum*, and the other failed bioinformatics QC due to insufficient coverage (Supplementary Data Sheet 1).

Of the 28 samples, 53.6% (15) were from women and 46.4% (13) were from men. The median age was 39 years, with an interquartile range (IQR) of 28 to 48. Most of the samples came from the Waterberg and Mopani districts of Limpopo province, accounting for 28.6% (8/28) and 25% (7/28), respectively. The Sekhukhune district had the lowest representation with 7.1% (2/28) of the samples (Supplementary Table 2).

The isolates were confirmed to be drug resistant with varying degrees of resistance revealed by WGS. All isolates had resistance to rifampicin. High mutations were observed in strains of the *rpo*B gene in p. Ser450Leu 39% (11/28). Isolates with resistance to Isoniazid were 61% (17/28) with *kat*G p. Ser315Thr 59% (10/17) representing most mutations. FLQ resistance was found in 54% (15/28) and most mutations in gyrA p. Asp94Gly were found in 47% (7/15). BDQ resistance-confining mutations were found in 18% (5/28) of the isolates with 40% (2/5) with a mutation in *mmp*R5 c.198dupG and 40% (2/5) with the mmpR5 c.198delG mutation. Isolates conferring resistance to BDQ also had resistance to clofazimine with a similar mutation pattern. Only 3.6% (1/28) had resistance to linezolid and none to BDQ. No mutations for delamanid and pretomanid were observed (Supplementary Table 3). Of the 28 unique variants reported by MAGMA, only one was not listed in the WHO drug-resistant mutations catalogue.

Among the 28 isolates, 53.6% (15/28) were lineage 4 - Latin American Mediterranean (LAM) strain, 35.7% (10/28) were lineage 2 – Beijing strain, 3.6% (1/28) was lineage 1 –Indo-Oceanic, 3.6% (1/28) was lineage 3 East African-Indian (EAI) and 3.6% (1/28) had mixed lineages 2 and 4 (Supplementary Table 4).

Lineages 2 and 4 were predominant in the Waterberg district and mixed lineage strains were from the Vhembe district (Supplementary Table 5). Lineages 2 and 4 had the most resistant genotypes, with all Pre-XDR-TB and XDR-TB genotypes observed in these two lineages. Resistance to linezolid was only observed in Lineage 4, which was more likely to be resistant to rifampicin, isoniazid, ethambutol, pyrazinamide, streptomycin, and linezolid than in Lineage 2 (*p* < 0.05). Furthermore, complicated drug-resistant cases were more likely to be present in lineage 4 (*p* < 0.001).

**Figure 1:**
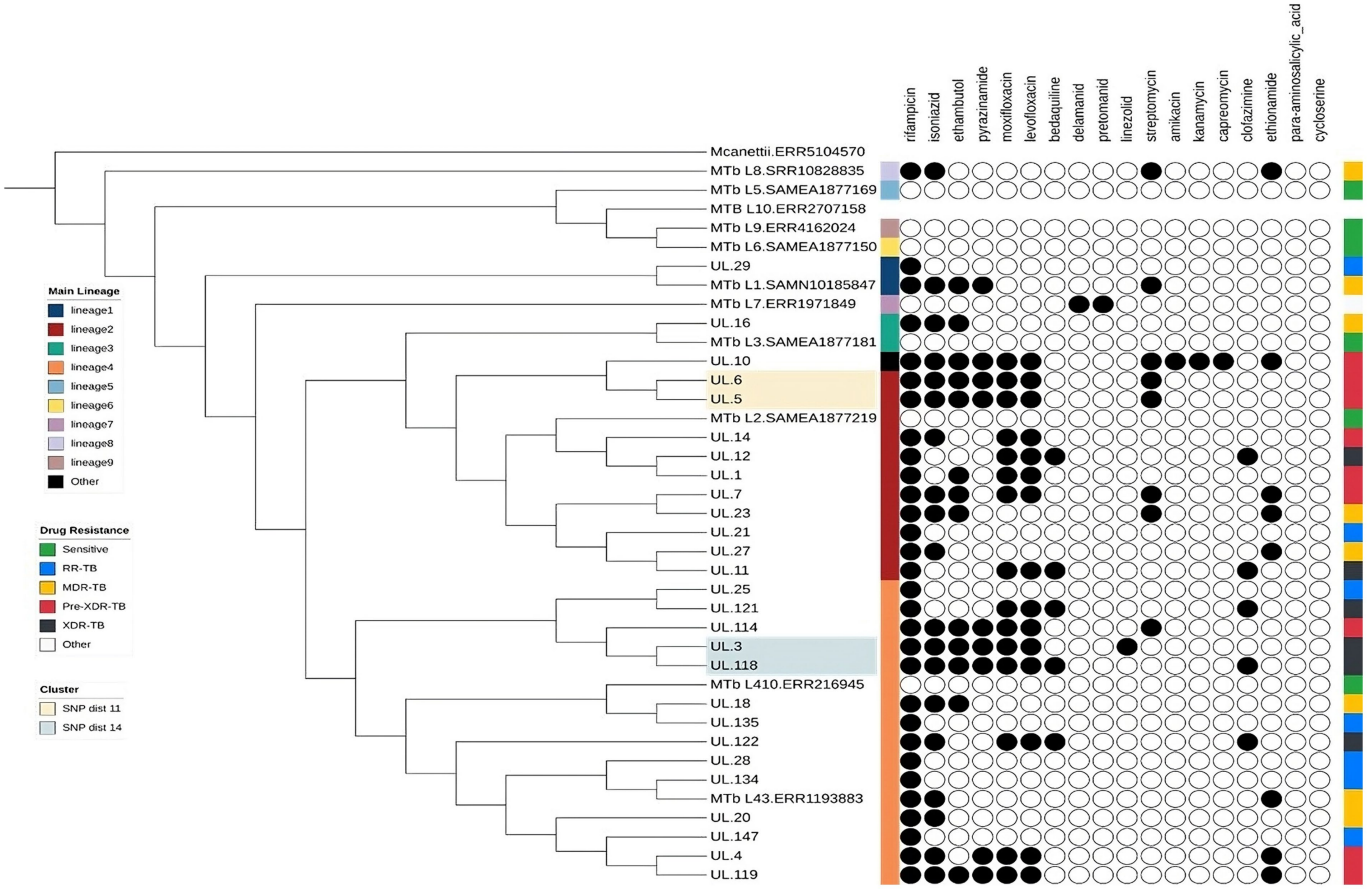
Phylogenetic tree of Mycobacterium tuberculosis isolates from Limpopo province, South Africa, with representative lineages (L1–L10), annotated by lineage and predicted drug resistance.

Transmission analysis revealed two distinct clusters of isolates. One cluster consisted of Pre-XDR isolates belonging to Lineage 2.2.2, while the other cluster included both Pre-XDR and XDR isolates from Lineage 4.1.1. The SNP distances were 11 and 14 SNPs, respectively (Figure 1).

Odds ratio analysis was conducted to examine the association between clustering status and demographic characteristics. Clustered cases were slightly more common among patients aged 16–35 years (OR = 1.45, 95% CI: 0.38–5.52, *p* = 0.58) and among females (OR = 1.29, 95% CI: 0.34–4.91, *p* = 0.70). However, these associations were not statistically significant (*p* > 0.05), indicating that clustering was not significantly associated with age or gender in this dataset (Supplementary Table 6).

## Discussion

Our capacity-building efforts led to the initial genomic analysis of 28 *Mtb* DR isolates from Limpopo province. These isolates were comprehensively characterized, paving the way for expanded genomic surveillance of TB in this key border region of South Africa. Due to the limited genetic diversity data available for *Mtb* in Limpopo, this study highlights the need for further characterization in the region.

High levels of drug resistance were found in the isolates by WGS. All isolates showed resistance to RIF, while 61% of isolates showed resistance to INH. The majority of resistance to RIF (39.3%) was due to the *rpo*B Ser450Leu mutation, while the majority of resistance to isoniazid (INH) (60.7%) was due to the *kat*G Ser315Thr mutation. These findings are consistent with other studies that reported that most of the isolates of resistance to RIF and INH were due to rpoB Ser450Leu and *katG* Ser315Thr, respectively (9, 10, 19). Noorizhab et al. proposed that the close link between these mutations and drug resistance indicates that they are reliable indicators of resistance to Iisoniazid and rifampicin (9). Resistance to rifampicin and INH is a major concern, as it is a cornerstone of TB treatment regimens. The widespread occurrence of a high level of resistance poses a serious threat to TB management strategies for robust strategies to monitor and manage drug resistance.

An alarming percentage of 54% of the isolates were found to be resistant to FLQ considering the importance of FLQ as a second-line treatment for DR-TB. The reduced efficacy of FLQs, which are frequently used as second-line treatments for drug-resistant tuberculosis, greatly complicates treatment regimens. Most of the resistance to FLQ was due to *gyrA* Asp94Gly (46.7%) followed by *gyrA* Ala90Val (33.3%). The results of this study were found to be similar to the results reported in other studies, where Asp94Gly (associated with high-level resistance) and Ala90Val (linked to low-level resistance) were the first and second most common mutations identified in both groups (20, 21). Thus, the current study appears to suggest a consistence of alleles associated with low- or high-level resistance of FLQ.

We report that 17.9% of the resistant associated variants (RAVs) to BDQ were due to the presence of the *mmpR5* gene mutation alone. The transcriptional repressor protein *mmpR5* is encoded by the *Rv0678* gene and has been reported to have cross-resistance to clofazimine. The detection of BDQ RAVs in this study further emphasizes the significant clinical challenge previously reported. Consistent with the findings, previous studies have indicated that resistance to BDQ is primarily attributed to Rv0678 (22, 23). While this study provides valuable insights into the genotypic resistance patterns *of M. tuberculosis* in Limpopo using WGS, it is important to acknowledge the absence of pDST data. This limits our ability to validate the clinical significance of some mutations, particularly those associated with newer or repurposed drugs such as BDQ and clofazimine. Nevertheless, the study employed the MAGMA pipeline, which annotates variants based on the WHO-endorsed catalogue of resistance-associated mutations, thereby enhancing the reliability of genotypic predictions. Despite this, we recognize that not all mutations, especially in genes such as mmpR5, have well-established phenotypic correlations. Future studies integrating WGS with pDST will be essential to refine our understanding of the functional implications of these mutations and strengthen clinical decision-making in drug-resistant TB management.

A study conducted in South Africa reported *rv0678* as the most common cause of resistance to BDQ (23). However, in another ongoing longitudinal cohort of drug-resistant samples from Limpopo, another RAVs of BDQ was reported due to the mutation in the atpE gene and could suggest the possibility of more RAVs present in Limpopo province and the need for continued surveillance of drug resistance of BDQ, as BDQ now forms part of the standard regimen for the treatment of DR-TB. Bedaquiline is a relatively new antibiotic used specifically for the treatment of MDR-TB and XDR-TB. Increased resistance to this drug threatens the effectiveness of current TB treatment regimens and complicates efforts to control and eradicate the disease. The study reports that resistance mutations to the delamanid and pretomanid were not detected.

In the current study, a rich tapestry of *Mtb* diversity was observed. In general, four main lineages were identified, including lineage 4 -Latin-American Mediterranean (LAM) strain 2 – Beijing strain, lineage 1 –Indo-Oceanic and lineage 3 East African-Indian (EAI), and 3.6% (11/28) had mixed lineages 2. The diverse distribution is expected, as Limpopo has major border gates that provide direct linkage with neighboring countries, and this may influence the genetic diversity of Mtb isolates in the province. Additionally, Limpopo province is predominantly agricultural, with significant populations of seasonal and migrant workers, and is known for its mining activities. The high mobility and the large number of young single job seekers contribute to the elevated rates of diversity in TB among farm workers in the province (24).

Lineage 4 (Latin American-Mediterranean) emerged as the dominant strain in our study, accounting for 53.6% of the isolates. This lineage is prevalent in Europe, the United States, and parts of Africa and is one of the most widely distributed lineages globally. This finding aligns with observations reported in southern Africa and Africa in general (South Africa, Mozambique, Zimbabwe, Zambia and Malawi) loosely grouped with Northern and Western African countries (Algeria, Morocco, and Guinea Bissau) (AU value 86%) based on the high percentage of LAM in these regions who reported a high prevalence of Lineage 4 in Africa along with other studies (9, 25, 26). Lineage 4 is known to drive global infection by *Mtb* and is also highly transmissible (9, 15). Lineage 4 has been more frequently associated with MDR-TB and XDR-TB (27). Furthermore, studies conducted in South Africa in the Free State province also found a high prevalence of LAM strains (64%) (27, 28). This geographic proximity suggests the potential for shared transmission pathways for LAM strains in South Africa.

The second most prevalent lineage in our study was lineage 2 (Beijing). Alarmingly, Lineage 2 in the current study exhibited a significantly higher percentage of isolates with pre-XDR-TB. This aligns with the observations of Maguga-Pasha et al., which highlighted the continued presence and potential threat of drug-resistant Beijing strains in Limpopo Province (5). Similarly, another study conducted in Beijing city found that 81.7% of *Mtb* strains were of the Beijing genotype, representing a predominant lineage with a high clustering rate (29). The presence of Pre-XDR-TB within a prevalent lineage raises significant public health concerns. Early detection and appropriate treatment regimens are even more crucial in preventing progression to full XDR-TB and curbing its spread.

The findings of this study should be interpreted within the broader epidemiological context of drug-resistant tuberculosis in South Africa. National estimates indicate that approximately 3.5% of new TB cases and 18% of previously treated cases are rifampicin-resistant, with growing concern about emerging resistance to FLQ and BDQ, the cornerstone drugs in current DR-TB regimens (2, 6). The identification of mutations conferring resistance to these critical agents in our cohort underscores the clinical relevance of genomic surveillance, particularly in under-resourced and high-mobility settings such as Limpopo. Although this study was limited by the absence of detailed clinical data (e.g., treatment outcomes, HIV status), due to its retrospective laboratory-based design, future studies should integrate genomic, clinical, and treatment data to guide individualized therapy and public health interventions.

Our analysis revealed two distinct putative clusters of *Mtb* strains L2 and L4. A group consisted of isolates exclusive to the Capricorn district, suggesting localized transmission within the area. In contrast, the other group contained isolates from two geographically distinct districts (Mopani and Capricorn), indicating the possibility of interdistrict transmission throughout the Limpopo province. Studies in Limpopo and the Free State provinces have previously reported similar transmission dynamics (5, 30). These findings highlight the ongoing circulation of *Mtb* within the province and suggest various transmission patterns. However, the two putative groups were identified from 2 samples and these groups may need to be confirmed with a larger sample size. Interestingly, studies in Nigeria and China also reported a higher clustering of L2 and L4 (9, 31). This further supports the notion that both L2 and L4 are highly transmissible (9). Furthermore, our identification of localized and inter-district transmission is in line with the findings of Maguga-Pasha, whose study identified 41 clusters within districts, further supporting the idea of ongoing and geographically diverse transmission of MTB in Limpopo province (5), which warrants a more strategic approach to the care and management of patients with DR-TB to limit further transmission.

Demographic information shows that, with a median age of 39 years (IQR: 28–48), women comprised the majority of patients (53.6%). To customize therapies, these demographic data are essential. The prevalence of TB from woman to man may be a sign of underlying health or socioeconomic inequalities that need to be addressed.

The samples were from all districts of the province, with Waterberg and Mopani dominant. Most (32%) of the waterberg genomic variants are attributable to the fact that the MDR-TB referral units of the Limpopo Province unit are located in the Waterberg district at the Modimolle facility. The Mopani district had the second highest incidence with 25% genomic variants. The Waterberg and Mopani district has a mining industry, as well as an agricultural and tourism area, providing the main employment for the migrant and immigrant populations. Due to the high rates of mobility in the area and the high proportion of people looking for work, the studies carried out in the district have revealed that farm workers have high rates of TB and HIV infections and have further stressed the stimulation of these socioeconomic factors on the spread of TB (26, 32). The fact that most of the samples came from the Mopani and Waterberg districts raises the possibility that these places are TB transmission hotspots. To manage the spread of DR-TB, these districts could be the target of public health initiatives. The low representation in the Sekhukhune district suggests that there may be fewer or under-reported instances, which calls for investigation to ensure comprehensive surveillance.

The samples were from the five districts of the province, with Waterberg and Mopani dominant. Most (32.2%) of the Waterberg genomic variants can be attributed to the fact that the MDR-TB unit is in Modimolle within the Waterberg district. The Mopani district had the second highest incidence with 25% genomic variants. Both the Waterberg and Mopani districts have a significant mining industry, as well as the agricultural and tourism sectors, providing the main employment for the migrant and immigrant populations. Due to the high rates of mobility in the area and the high proportion of people seeking employment, studies conducted in these districts have revealed that farm workers have elevated rates of TB and HIV infections, further emphasizing the impact of these socioeconomic factors on the spread of TB (33, 34).

The limitation of the study was related to the limited resources required to perform WGS on all samples (*n* = 232). To gain a more complete understanding of clustering rates and possible links between treatment and the emergence of DR strains, future studies may include a larger number of samples and detailed clinical information. However, longitudinal studies may also provide invaluable insights into the evolutionary trends. We propose.

### Limitations

The sample size was limited to 30 isolates due to financial and logistical constraints, which may affect the generalizability of the findings. A formal power analysis was not conducted, as the primary aim was exploratory, to initiate genomic surveillance and characterize resistance-associated mutations using WGS in a resource-limited setting. As such, the results should be interpreted with caution and viewed as foundational. Additionally, the absence of pDST limited our ability to validate the clinical relevance of some resistance mutations, particularly those related to newer drugs such as BDQ and clofazimine. Due to the relatively small sample size and exploratory nature of this study, multiple testing corrections were not applied when comparing across districts and lineages. This may increase the risk of false-positive findings, and results should therefore be interpreted with caution. Future studies with larger, systematically selected cohorts and integrated phenotypic-genotypic data will be essential to build on these findings.

## Conclusion

In conclusion, this study provides evidence that the TB epidemic in Limpopo province involves a diverse range of *Mycobacterium tuberculosis* lineages, with significant genetic variation also observed at the district level. The predominant strains identified belonged to the LAM genotype lineages L4 and L2 (Beijing strains), which are commonly associated with drug-resistant TB globally. The identification of genetically related strains within the same laboratory setting may suggest possible nosocomial or community-based transmission, although this inference is limited by the absence of direct epidemiological linkage data.

While a potential association between specific lineages and drug resistance was observed, further research incorporating larger sample sizes and detailed clinical data is needed to confirm this relationship. These findings highlight the importance of considering the genetic characteristics of circulating strains in the province to inform TB control strategies.

## Data Availability

The datasets presented in this study can be found in online repositories. The raw fastq files were submitted to the National Center for Biotechnology Information (NCBI) under the BioProject ID PRJNA1215970, Biosamples ID from SAMN46422214 to SAMN46422242.

## Footnotes

^1^https://mstrweb.nicd.ac.za/MicroStrategy/asp/Main.aspx, accessed October 23, 2024.

^2^ https://www.bioinformatics.babraham.ac.uk/projects/fastqc

^3^ http://broadinstitute.github.io/picard

^4^ https://itol.embl.de
