# Supplementary material for: Empowering tuberculosis genomic surveillance in Limpopo, South Africa through capacity building
Source: Front Public Health. 2025 Sep 12;13:1567382. doi: 10.3389/fpubh.2025.1567382 (PMC12463882; doi:10.3389/fpubh.2025.1567382)
Supplement: Supplementary file 2 [file Table_1.docx]

**Supplementary Table 1.** Training course program on tuberculosis genetics.

| **Day** | **Period** | **Activity** |
| --- | --- | --- |
| 1 | Morning | Presentation and practice of the course using the Unix system |
|  | Afternoon | Lecture: Introduction to Next-Generation Sequencing |
|  |  | Lecture: DNA extraction and quality control (QC) - Mtb |
|  |  | Practice: DNA extraction using IGM/FP and QC (controls) |
| 2 | Morning | Lecture: Library Preparation and QC |
|  | Afternoon | Lecture: Library Preparation Calculations |
| 3 | Morning | Lecture: Overview on Tuberculosis Genomics: From Genotyping to Next-Generation Sequencing |
|  | Afternoon | Lecture: Computers and Bioinformatic Pipelines Structure |
| 4 | Morning | Practice: DNA extraction using IGM/FP and QC |
|  | Afternoon | Practice: DNA extraction with samples from Limpopo |
| 5 | Morning | Practice: Library Preparation and QC |
|  | Afternoon | Practice: Library Preparation and QC |
| 6 | Morning | Practice: Library Preparation and Quality Control |
|  | Afternoon | Practice: Pooling and start sequencing |
| 7 | Morning | Practice: MAGMA pipeline overview and sample sheet preparation |
|  | Afternoon | Practice: MAGMA pipeline execution (published samples) |
| 8 | Morning | Practice: Execution of the MAGMA pipeline (Limpopo Samples) |
|  | Afternoon | Diplomacy Opportunities |
|  |  | Visit to SAMRC |
| 9 | Morning | Data-interpretation and presentation preparation |
|  | Afternoon | Data Interpretation and Presentation Preparation |
| 10 | Morning | Present the results within the TB Genomics lab meeting |
