# Supplementary material for: Empowering tuberculosis genomic surveillance in Limpopo, South Africa through capacity building
Source: Front Public Health. 2025 Sep 12;13:1567382. doi: 10.3389/fpubh.2025.1567382 (PMC12463882; doi:10.3389/fpubh.2025.1567382)
Supplement: Supplementary file 3 [file Table_2.docx]

**Supplementary Table 2. Demographic characteristics of drug-resistant tuberculosis patients**

| **Variable** | | **RR-TB *n*(%)** | **MDR-TB *n*(%)** | **Pre-XDR-TB *n*(%)** | **XDR-TB *n*(%)** | **P-value** | **Total *n*(%)** |
| --- | --- | --- | --- | --- | --- | --- | --- |
| **Age** | 16-35 | 2 (15.4) | 4 (30.8) | 2 (15.4) | 5 (38.5) | 0.161 | 13 (46.4) |
|  | 36-55 | 5 (38.5) | 2 (15.4) | 5 (38.5) | 1 (7.8) |  | 13 (46.4) |
|  | >55 | 0 (0) | 0 (0) | 2 (100) | 0 (0) |  | 2 (7.1) |
| **Gender** | Male | 4 (30.8) | 2 (15.4) | 5 (38.5) | 2 (15.4) | 0.610 | 13 (46.4) |
|  | Female | 3 (20) | 4 (26.7) | 4 (26.7) | 4 (26.7) |  | 15 (53.6) |
| **District** | Capricorn | 1 (16.7) | 1 (16.7) | 1 (16.7) | 3 (50) | 0.740 | 6 (21.4) |
|  | Waterberg | 2 (25) | 2 (25) | 2 (25) | 2 (25) |  | 8 (28.6) |
|  | Mopani | 3 (42.9) | 2 (28.6) | 2 (28.6) | 0 (0) |  | 7 (25) |
|  | Vhembe | 1 (20) | 1 (20) | 2 (40) | 1 (20) |  | 5 (17.9) |
|  | Sekhukhune | 0 (0) | 0 (0) | 2 (100) | 0 (0) |  | 2 (7.1) |
| **Year** | 2021 | 3 | 0 (0) | 0 (0) | 0 (0) | 0.666 | 3 (10.7) |
|  | 2022 | 0 (0) | 0 (0) | 2 (40) | 3 (60) |  | 5 (17.9) |
|  | 2023 | 4 (20) | 6 (30) | 7 (35) | 3 (15) |  | 20 (71.4) |

^RR-TB- resistant to rifampicin, MDR-TB- multidrug resistant TB, Pre-XDR-TB- pre-extensively drug resistant TB, and XDR-TB extensively drug resistant TB.^
