# Supplementary material for: Empowering tuberculosis genomic surveillance in Limpopo, South Africa through capacity building
Source: Front Public Health. 2025 Sep 12;13:1567382. doi: 10.3389/fpubh.2025.1567382 (PMC12463882; doi:10.3389/fpubh.2025.1567382)
Supplement: Supplementary file 4 [file Table_3.docx]

**Supplementary Table 3. Drug resistance gene mutation spectrum of the 28 sequenced isolates.**

|  | | **MAGMA** | | | **WHO Catalogue (reference)** | |
| --- | --- | --- | --- | --- | --- | --- |
| **Drug** | **Resistant strains (%)** | **Variant** | **No. of mutant strains** | **Resistant or Sensitive Annotation** | **Variant** | **Resistant or Sensitive Annotation** |
| Rifampicin | **28 (100)** | *rpoB p.Ser450Leu*  *rpoB p.Leu452Pro*  *rpoB p.Asp435Val*  *rpoB p.His445Tyr*  *rpoB p.Asp435Tyr*  *rpoB p.His445Asp*  *rpoB p.Leu430Pro*  *rpoB p.His445Asn* | 11 (39.3%)  4 (14.3%)  3 (10.7%)  3 (10.7%)  2 (7.1%)  2 (7.1%)  2 (7.1%)  1 ( 3.6%) | R  R  R  R  R  R  R  R | *rpoB p.Ser450Leu*  *rpoB p.Leu452Pro*  *rpoB p.Asp435Val*  *rpoB p.His445Tyr*  Not listed  *rpoB p.His445Asp*  *rpoB p.Leu430Pro*  *rpoB p.His445Asn* | R  R  R  R  R  R  R |
| Isoniazid | **17 (60.7)** | *katG p.Ser315Thr*  *inhA c.-770T>A plus katG p.Ser315Thr*  *inhA c.-154G>A*  *inhA c.-777C>T*  *inhA c.-779G>T plus katG p.Ser315Thr*  *katG c.18_27delACCCATTACA*  *katG p.Ser315Arg* | 10 (58.8%)  2 (11.8%)  1 (5.9%)  1 (5.9%)  1 (5.9%)  1 (5.9%)  1 (5.9%) | R  R  R  R  R  R  R | *katG p.Ser315Thr*  *inhA c.-770T>A plus katG p.Ser315Thr*  *inhA c.-154G>A*  *inhA c.-777C>T*  *inhA c.-779G>T plus katG p.Ser315Thr*  Not listed.  *katG p.Ser315Arg* | R  R  R  R  R  R |
| Fluoroquinolones | **15 (53.6)** | *gyrA p.Asp94Gly*  *gyrA p.Ala90Val*  *gyrA p.Asp94Ala*  *gyrA p.Ala90Val plus gyrA p.Asp94Ala* | 7 (46.7%)  5 (33.3%)  2 (13.3%)  1 (6,7%) | R  R  R  R | *gyrA p.Asp94Gly*  *gyrA p.Ala90Val*  *gyrA p.Asp94Ala*  *gyrA p.Ala90Val plus gyrA p.Asp94Ala* | R  R  R  R |
| Bedaquline | **5 (17.9)** | *mmpR5 c.198dupG*  *mmpR5 c.198delG*  *mmpR5 c.144dupC* | 2 (40%)  2 (40%)  1 (20%) | R  R  R | *mmpR5 c.198dupG*  *mmpR5 c.198delG*  *mmpR5 c.144dupC* | R  R  R |
| Linezolid | **1 (3.6)** | *rplC p.Cys154Arg* | 1 (100%) | R | *rplC p.Cys154Arg* | R |
