# Supplementary material for: Empowering tuberculosis genomic surveillance in Limpopo, South Africa through capacity building
Source: Front Public Health. 2025 Sep 12;13:1567382. doi: 10.3389/fpubh.2025.1567382 (PMC12463882; doi:10.3389/fpubh.2025.1567382)
Supplement: Supplementary file 5 [file Table_4.docx]

**Supplementary Table 4. Lineage Characteristics Explicit by WGS**

| **Genotypic Characteristics** | | **Lineage 1 Indo-Oceanic strains** | **Lineage 2 East Asian/ Beijing strain** | **Lineage 3 East African-Indian strain** | **Lineage 4 Latin-American Mediterranean (LAM) strain** |
| --- | --- | --- | --- | --- | --- |
| **No. of genotypic variants identified** | | 1 genotypic DR variant | 10 genotypic DR variants | 1 genotypic DR variant | 15 genotypic DR variants |
| **Dominant drug resistance profile** | | RR-TB | Pre-XDR and XDR-TB | MDR-TB | Pre-XDR and XDR-TB |
| **Dominant Mutation Type** | **RIF** | -*rpoB* p.Leu430Pro | -*rpoB* p.Ser450Leu | - *rpoB* p.His445Tyr | *-rpoB* p.Ser450Leu |
|  | **INH** | - No mutation | *-katG* p.Ser315Thr | *- katG* p.Ser315Thr | *-katG* p.Ser315Thr |
|  | **FLQs** | - No mutation | *-gyrA* p.Asp94Glyd | - No mutation | - *gyrA* p.Ala90Val |
|  | **BDQ and CFZ** | - No mutation | - only  *mmpR5* c.198delG detected | - No mutation | *- mmpR5* c.198dupG |
|  | **LZD** | - No mutation | No mutation | - No mutation | *- rplC* p.Cys154Arg |

^RIF-Rifampicin, INH- Isoniazid, FLQs- Fluroquinolones, BDQ- Bedaquiline, CFZ-Clofazamine, LZD-Linezolid^
