# Supplementary material for: Empowering tuberculosis genomic surveillance in Limpopo, South Africa through capacity building
Source: Front Public Health. 2025 Sep 12;13:1567382. doi: 10.3389/fpubh.2025.1567382 (PMC12463882; doi:10.3389/fpubh.2025.1567382)
Supplement: Supplementary file 6 [file Table_5.docx]

**Supplementary Table 5. Lineage distribution by DR-TB type and district municipality in Limpopo**

| **Variables** | | **Lineage 1** | **Lineage 2** | **Lineage 3** | **Lineage 4** | **Lineage 2 & 4** | **Total** |
| --- | --- | --- | --- | --- | --- | --- | --- |
| DR-TB Type | RR-TB | 1 (3.6) | 1 (3.6) | - | 5 (17.9) | - | 7 (25.0) |
|  | MDR-TB | - | 2 (7.2) | 1 (3.6) | 3 (10.7) | - | 6 (21.4) |
|  | PreXDR-TB | - | 5 (17.9) | - | 3 (10.7) | 1 (3.6) | 9 (32.2) |
|  | XDR-TB | - | 2 (7.2) | - | 4 (13.8) | - | 6 (21.4) |
| District | Capricorn | - | 3 (10.7) | - | 2 (7.1) | - | 5 (17.8) |
|  | Waterberg | - | 4 (14,3) | - | 5 (17.9) | - | 9 (32.2) |
|  | Mopani | 1 (3.6) | 2 (7.1) | 1 (3.6) | 3 (10,7) | - | 7 (25.0) |
|  | Vhembe | - | 0 (0) | - | 4 (14.3) | 1 (3.6) | 5 (17.8) |
|  | Sekhukhune | - | 1 (3.6) | - | 1 (3.6) | - | 2 (7.2) |
