# Supplementary material for: Empowering tuberculosis genomic surveillance in Limpopo, South Africa through capacity building
Source: Front Public Health. 2025 Sep 12;13:1567382. doi: 10.3389/fpubh.2025.1567382 (PMC12463882; doi:10.3389/fpubh.2025.1567382)
Supplement: Supplementary file 7 [file Table_6.docx]

**Supplementary Table 6. Fisher’s exact comparison of drug-resistant profiles between lineage 2 and lineage 4**

| **Drugs/ drug resistant patterns** | | **Lineage 2**  **(n=10)**  **n(%) [95% CI]** | **Lineage 4**  **(n=15)**  **n (%) [95% CI]** | **P-value** |
| --- | --- | --- | --- | --- |
| **First-line drugs** | Rifampicin | 10 (100) [72.2-100%] | 15 (100) [72.2-100%] | NA |
|  | Isoniazid | 6 (60) [31.3-83.2%] | 9 (60) [35.7-80.2%] | 1.00 |
|  | Streptomycin | 4 (40) [35.7-80.2%] | 1 (6.6) [1.2-29.8%] | 0.046 |
|  | Ethambutol | 5 (50) [23.7-76.3%] | 5(33.3) [15.0-58.5%] | 0.453 |
|  | Pyrazinamide | 2 (20) [5.7-51.0%] | 5 (33.3) [15.0-58.5%] | 0.669 |
| **Second-line drugs** | Levofloxacin | 7 (70) [39.7-89.2%] | 7 (46.7) [24.8-69.9%] | 0.262 |
|  | Moxifloxacin | 7 (70) [39.7-89.2%] | 7 (46.7) [24.8-69.9%] | 0.262 |
|  | Bedaquiline | 2 (20) [5.7-51.0%] | 3 (20) [7.1-45.2%] | 1.000 |
|  | Clofazamine | 2 (20) [5.7-51.0%] | 3 (20) [7.1-45.2%] | 1.000 |
|  | Linezolid | 0 (0) [0-27.8%] | 1 (6.6) [1.2-29.8%] | 1.000 |
| **DR- profile** | RR-TB | 1 (10) [1.8-40.4%] | 5 (33.3) [15.0-58.5%] | 0.214 |
|  | MDR-TB | 2 (20) [5.7-51.0%] | 3 (20) [7.1-45.2%] | **1.000** |
|  | PreXDR-TB | 5 (50) [23.7-76.3%] | 3 (20) [7.1-45.2%] | **0.113** |
|  | XDR-TB | 2 (20) [5.7-51.0%] | 4 (26.7) [10.9-52.0%] | **1.000** |
